# ATP-binding cassette transporters in immortalised human brain microvascular endothelial cells in normal and hypoxic conditions

**DOI:** 10.1186/2040-7378-4-9

**Published:** 2012-05-03

**Authors:** Christian Lindner, Alexander Sigrüner, Franziska Walther, Ulrich Bogdahn, Pierre O Couraud, Gert Schmitz, Felix Schlachetzki

**Affiliations:** 1 Department of Neurology, University of Regensburg, Bezirksklinikum Regensburg, Regensburg, Germany; 2 Department of Psychiatry, University of Regensburg, Bezirksklinikum Regensburg, Regensburg, Germany; 3 Institute of Clinical Chemistry and Laboratory Medicine, Regensburg University Medical Center, Regensburg, Germany; 4 Departement de biologie cellulaire (CNRS UMR 8104), Institut Cochin, Université Paris Descartes, Paris, France and INSERM U567, Departement de biologie cellulaire, Institut Cochin, Paris, France

**Keywords:** Blood–brain barrier, Ischemia/reperfusion injury, Hypoxia-inducible factor, Multidrug resistance, ABC transporters, Stroke

## Abstract

**Background:**

Rapid reperfusion following ischemia is the most effective therapy in stroke therapy. However, the success may be compromised by ischemia & reperfusion (I/R) injury and at the human blood–brain barrier (BBB), therefore the effects on transendothelial transport are of special interest. Current studies suggest the ATP-binding cassette (ABC) transporters to be regulated upon ischemic stroke in a way that impedes the effects of drug therapy. The immortalised human brain microvascular endothelial cell line hCMEC/D3 provides most of the unique properties of the BBB with respect to transport and might be a reliable in vitro model to study transendothelial transport after I/R.

**Methods:**

We exposed hCMEC/D3 cells to 24 hours of hypoxia alone and to hypoxia followed by 60 min of reoxygenisation as an in vitro model for I/R. Western blot showed mild upregulation of hypoxia inducible factor (HIF-1α) after hypoxia alone and RNA lysates were analysed with a well-established real-time RT-PCR-based TaqMan low-density array detecting 47 of 48 known human ABC transporters.

**Results:**

No significant increases of ABC mRNA expression levels were detected neither in hypoxic nor in I/R samples. However, slight decrease of ABCC1 in hypoxic and I/R samples and of ABCA10 and ABCD3 in I/R samples was observed.

**Conclusion:**

Our data suggests that hCMEC/D3 cell line and – at the moment – in vitro models in general are a poor basis for stroke research but may be enhanced by co-culturing more cells of the neurovascular unit inducing an overall ischemic response at the BBB.

## Background

The successful rescue of penumbral brain tissue by rapid reperfusion may be compromised by ischemia/reperfusion injury (I/R) and other secondary events, amongst them post-ischemic inflammatory response, excitotoxicity, excess of reactive oxygen species (ROS), and induction of apoptotic neuronal cell death [1-3]. The cerebral endothelium, which forms the blood–brain barrier (BBB) in-vivo, may play a crucial role in post-ischemic reperfusion for several reasons: 1.) it is the primary site where reperfusion occurs, 2.) it allows interaction between the brain’s and body’s immune system and, 3.) it strongly interacts with other cell types of the neurovascular unit via cell-cell, cell-matrix and neuro-endocrine cross talk, amongst others, determining the overall cerebral response to ischemia [4-6]. Several studies with I/R stroke models demonstrated a dynamic, even biphasic BBB permeability increase, whereas in clinical stroke neurology only early post-ischemic BBB disruption has been associated with life threatening oedema formation and increased risk of symptomatic intracerebral hemorrhage [7-10]. However, other than BBB tight junction integrity several other BBB functions may be compromised and contribute to I/R injury. Especially transendothelial transport is of special interest for I/R injury and may influence brain nutrition, systemic response to brain ischemia, and even drug therapy of the brain [11,12].

Drug therapy is compromised by endothelial efflux transporters normally responsible for brain detoxification, and the best-characterised efflux transporters of the brain’s endothelium are p-glycoprotein (P-gp, MDR1, ABCB1) and breast cancer resistance protein (BCRP, ABCG2) [11,13,14]. Both belong to the ATP-binding cassette (ABC) transporter family, which comprises 48 multispan membrane proteins and is further divided into seven subfamilies (from ABCA to ABCG) according to sequence homology [15]. ABC transporters either promote or regulate the transport of specific substrates across various biological membranes, including sugars, amino acids, metal ions, peptides, and proteins as well as a large number of hydrophobic compounds and metabolites, and are abundantly expressed at the BBB [16-19]. Moreover, several ABC-transporters at the BBB play an important role in multi-drug resistance thereby offering brain protection from toxic compounds by acting as efflux transporters, and unfortunately for several drugs causing multidrug resistance [11,19,20].

Only few studies investigate the regulation of ABC-transporters upon ischemia, I/R and regeneration. In vivo studies suggest that the expression of ABC transporters is upregulated upon ischemia, impeding the delivery of drugs into the brain [21,22]. A recent study by Patak et al. investigated the regulation of ABCB1 and ABCC1 by hypoxia in immortalised human brain microvascular endothelial cells (hCMEC/D3), a very common and well characterised cell line for transport studies as unique properties of the BBB persist [23-26]. Surprisingly, Patak et al. found no changes of ABCB1 and ABCC1 expressions neither on the mRNA nor protein level immediately after hypoxia. The authors speculated that regulation of ABCB1 and ABCC1 as seen in cerebral ischemia could depend on other factors than hypoxia, such as glucose deprivation or reoxygenation.

In this study we characterise the immortalised human cerebral endothelial cell line hCMEC/D3 performing profiling of mRNA expression of 47 human ABC transporters and compared mRNA expression during normoxia, after 24 hours of hypoxia and after 24 hours of hypoxia followed by 60 min of reoxygenation resembling I/R with a reference panel from a large variety of other human tissues (Human universal reference total RNA). For the analysis, we used a well-established real-time RT-PCR-based TaqMan low-density array (TLDA) [27,28] and compared transcript levels of hypoxic and I/R samples to normoxic samples.

## Methods

### ***The hCMEC/D3 cell line***

The hCMEC/D3 cell line was developed by Weksler et al. by coexpressing hTERT and the SV40 large T antigen via a highly efficient lentiviral vector system in a primary cell culture from an epileptic region from the temporal lobe [23,29]. In brief, the goal was to establish a stable, fully characterised and well-differentiated human brain endothelial cell line which retains most of the unique properties of the BBB superior to other complex co-culture BBB models [30,31]. After several attemps [23,32-35] the hCMEC/D3 cell line was the first human brain endothelial cell line to retain most of the unique properties of the BBB without undergoing the rapid dedifferentiation and senescence characteristical for primary cultures of human brain endothelial cells.

### ***Cell culture***

hCMEC/D3 cells (passages 35–38) were grown in EBM-2 medium (Lonza, Verviers, Belgium) supplemented with hydrocortisone, ascorbate, gentamycin, VEGF, IGF-1, EGF, basic FGF and 2,5 % fetal calf serum as previously described [23] and called microvascular endothelial cell medium-2 (EGM-2MV). The cells were plated out in T75 flasks coated with type I collagen (PAN-Biotech, Aidenbach, Germany) and grown at 37°C in a humidified atmosphere of 5 % CO_2_. Cell flasks for ischemia and I/R used a vent/close closure and were placed in a hypoxia chamber with their closure slightly unclosed for 1 hour for equilibration at reduced oxygen level (oxygen concentration 3 %), then were completely closed and incubated for 23 hours. I/R samples were afterwards put back in a chamber at 37°C and humidified atmosphere of 5 % CO_2_ with their closure slightly unclosed to enable equilibration to normoxia for 60 min.

*RNA isolation, reverse transcription, Quantitative real-time PCR and Data analysis were performed as previously published*[27]*with the following differences:*

### ***RNA isolation***

For cell RNA isolation from cultured cells we used the RNeasy Mini Kit (Qiagen, Hilden, Germany) according to the manufacturer’s instructions.

### ***Data analysis***

Micro Fluidic Cards were analyzed with relative quantity (RQ) documents and the RQ Manager Software for automated data analysis. We performed 3 experiments for normoxic and hypoxic samples and two for I/R samples. Expression values for target genes were normalised to the concentration of 18 S rRNA. For each experiment, gene expression values were calculated on the basis of the comparative threshold cycle (*C*_t_) method, in which normoxic samples were designated as the calibrator to which the other samples were compared. In short, the *C*_t_ data for all human ABC transporters and 18 S rRNA in each sample were used to create ∆*C*_t_ values (*C*_tABC transporter_ – *C*_t18S rRNA_). For this calculation, the mean *C*_t_ over all experiments was used. Thereafter, ∆∆*C*_t_ values were calculated by subtracting the ∆*C*_t_ of the calibrator from the ∆*C*_t_ value of each target. The RQs were calculated using the equation: RQ = 2^-∆∆*C*t^. Genes that were regulated more than 2-fold (≥2.0 or ≤0.5) were considered as significantly regulated. The standard deviations (SD) for ∆*C*_t_ and ∆∆*C*_t_ values were calculated from the single *C*_t_ values with the equation: SD∆*C*_t_ = √(SD_tABC transporter_^2^ + SD_t18S rRNA_^2^). The ranges of the RQ values were calculated by use of the equations: RQ_min_ = 2^-∆∆*C*t-SD^ and RQ_max_ = 2^-∆∆*C*t+SD^.

### ***Antibodies***

The polyclonal goat antibody against human HIF-1α was purchased from Santa Cruz (sc-8711, Heidelberg, Germany). Molecular Weight of HIF-1α is 132 kDa. The donkey polyclonal, horse radish peroxidase (HRP)-conjugated antibody raised against goat IgG was purchased from Dianova (Hamburg, Germany).

### ***Western Blot***

Briefly, cell lysates were prepared using RIPA lysis buffer (50 mM Tris pH 7.4, 0.1 % SDS, 1 % Nonidet P40, 0.5 % sodium deoxycholate, 150 mM NaCl) containing protease inhibitor cocktail (Roche, Mannheim, Germany). Protein concentrations were quantified using a Bicinchoninic Acid-Assay. Protein samples were separated on 10 % NuPAGE Novex Bis-Tris gels (Invitrogen, Karlsruhe, Germany) and blotted onto PVDF membranes according to manufacturer’s instruction. After the transfer of 100 μg of each control and 24 hours hypoxia blocking of unspecific binding sites was achieved by incubation in TPBS (50 mM Tris/HCl, 150 mM NaCl) containing 0.1 % Tween 20 and 3 % skimmed milk. Washed membranes were incubated overnight at 4°C with antibody against HIF-1α (1:500 in 3 % skimmed milk). Following 1 hour incubation with the HRP-conjugated secondary anti-goat antibody (1:5000 in 3 % skimmed milk), the target protein was detected with the chemiluminescence HRP Substrate (Millipore, Schwalbach, Germany) using an X-Omat M35 Film Processor (Kodak, Stuttgart, Germany).

## Results

### ***Induction of HIF-1α under hypoxia***

**Figure 1:**
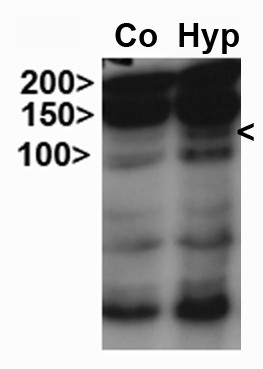
Upregulation of HIF-1 α Western blot after 24 hrs hypoxia compared to control at 130 kDa (right arrow).

After ischemia cell culture media showed a reddish colour indicating low pH as observed in hypoxia. To confirm hypoxia, cell lysates were analysed by Western blot to detect the protein expression of HIF-1α. HIF-1α was increased in a sample that underwent 24 hours of hypoxia as compared to the normoxic control (Figure 1). We conclude that our in vitro model of ischemia did indeed induce hypoxia in hCMEC/D3 samples.

### *Expression analysis of ABC-transporters in hCMEC/D3 cell line during normoxia*

To provide a profile of mRNA expression of human ABC transporters in the hCMEC/D3 cell line, expression levels were determined with TLDA technique. *C*_t_ values were compared to human universal reference calibrator RNA (huRNA) and ∆*C*_t_ values were calculated (means ± SD). Results are displayed as a dot code for high (●●●), medium (●●) and low (●) expressions, which are based on ∆*C*_t_ values (Table 1). For this analysis, the range between the lowest ∆*C*_t_ value and the highest ∆*C*_t_ value of genes expressed was divided linearly into three groups for high, medium and low expressions. Genes with not at least half of their *C*_t_ values below 35 cycles were defined as absent.

**Table 1:** Expression of ABC transporters in the immortalised human brain microvascular endothelial cell line hCMEC/D3

**Gene**	**Assay ID**	**Expression**	**Normoxic ΔC**_**t**_**(SD)**		**RQ (RQ Range)**	
ABCA1	Hs00194045_m1	●	16.31	(0.58)	0.65	(0.44-0.97)
ABCA2	Hs00242232_m1	●●	15.38	(0.54)	0.42	(0.29-0.62)
ABCA3	Hs00184543_m1	●●	14.83	(0.61)	7.85	(5.15-11.97)
ABCA4	Hs00184367_m1	●	17.21	(0.62)	2.20	(1.43-3.38)
ABCA5	Hs00363322_m1	●●●	12.27	(0.46)	6.54	(4.75-9.01)
ABCA6	Hs00365329_m1	●●●	12.82	(0.49)	22.92	(16.37-32.09)
ABCA7	Hs00185303_m1	●●	15.77	(0.47)	n.a.^a^	
ABCA8	Hs00200350_m1	●	17.90	(0.93)	0.31	(0.16-0.60)
ABCA9	Hs00329320_m1	●	17.94	(0.58)	0.66	(0.44-0.98)
ABCA10	Hs00365268_m1	●	17.04	(0.61)	1.30	(0.85-1.98)
ABCA12	Hs00292421_m1	n.e.^b^				
ABCA13	Hs00541549_m1	n.e.				
ABCB1	Hs00184491_m1	●●●	11.91	(0.40)	29.84	(22.55-39.48)
ABCB2	Hs00184465_m1	●●●	12.44	(0.40)	9.68	(7.31-12.81)
ABCB3	Hs00241060_m1	●●●	13.16	(0.43)	20.05	(14.90-26.98)
ABCB4	Hs00240956_m1	n.e.				
ABCB5	Hs00698751_m1	n.e.				
ABCB6	Hs00180568_m1	●●	14.08	(0.43)	2.62	(1.95-3.51)
ABCB7	Hs00188776_m1	●●●	13.47	(0.36)	6.02	(4.70-7.72)
ABCB8	Hs00185159_m1	●●	15.29	(0.46)	4.45	(3.23-6.13)
ABCB9	Hs00608640_m1	●●	15.71	(0.71)	0.92	(0.56-1.50)
ABCB10	Hs00429240_m1	●●	14.18	(0.37)	3.00	(2.33-3.87)
ABCB11	Hs00184824_m1	n.e.				
ABCC1	Hs00219905_m1	●●●	12.76	(0.47)	20.95	(15.10-29.07)
ABCC2	Hs00166123_m1	●	18.41	(0.43)	0.44	(0.33-0.59)
ABCC3	Hs00358656_m1	●●	14.44	(0.42)	8.23	(6.17-10.98)
ABCC4	Hs00195260_m1	●●●	12.49	(0.38)	8.64	(6.64-11.24)
ABCC5	Hs00194701_m1	●	17.44	(0.51)	3.06	(2.15-4.37)
ABCC6	Hs00184566_m1	n.e.				
ABCC7	Hs00357011_m1	n.e.				
ABCC8	Hs00165861_m1	n.e.				
ABCC9	Hs00245832_m1	n.e.				
ABCC10	Hs00375716_m1	●●	15.14	(0.45)	3.72	(2.72-5.10)
ABCC11	Hs00261567_m1	n.e.				
ABCD1	Hs00163610_m1	●	17.80	(0.77)	11.53	(6.77-19.64)
ABCD2	Hs00193054_m1	n.e.				
ABCD3	Hs00161065_m1	●●●	13.41	(0.36)	10.20	(7.94-13.11)
ABCD4	Hs00245340_m1	●●	14.87	(0.45)	4.59	(3.37-6.25)
ABCE1	Hs00759267_s1	●●●	11.80	(0.34)	10.10	(7.97-12.79)
ABCF1	Hs00153703_m1	●●	15.21	(0.44)	2.44	(1.80-3.31)
ABCF2	Hs00606493_m1	●●●	11.97	(0.47)	3.22	(2.33-4.46)
ABCF3	Hs00217977_m1	●●●	13.58	(0.32)	4.21	(3.36-5.27)
ABCG1	Hs00245154_m1	●●	15.48	(0.36)	1.05	(0.82-1.35)
ABCG2	Hs00184979_m1	●●	14.84	(0.50)	1.21	(0.85-1.72)
ABCG4	Hs00223446_m1	n.e.				
ABCG5	Hs00223686_m1	n.e.				
ABCG8	Hs00223690_m1	n.e.				

a) ABCA7 mRNA could not be detected in human reference RNA calibrator

b) n.e., not expressed

In cells of the hCMEC/D3 cell line we found no expression for ABCA12, ABCA13, ABCB4, ABCB5, ABCB11, ABCC6, ABCC7, ABCC8, ABCC9, ABCC11, ABCD2, ABCG4, ABCG5 and ABCG8. At least low levels of expression were found for ABCA1, ABCA4, ABCA8, ABCA9, ABCA10, ABCC2, ABCC5 and ABCD1, whereas medium levels were found for ABCA2, ABCA3, ABCA7, ABCB6, ABCB8, ABCB9, ABCB10, ABCC3, ABCC10, ABCD4, ABCF1, ABCG1 and ABCG2. High expression levels were found for ABCA5, ABCA6, ABCB1, ABCB2, ABCB3, ABCB7, ABCC1, ABCC4, ABCD3, ABCE1, ABCF2 and ABCF3.

**Figure 2:**
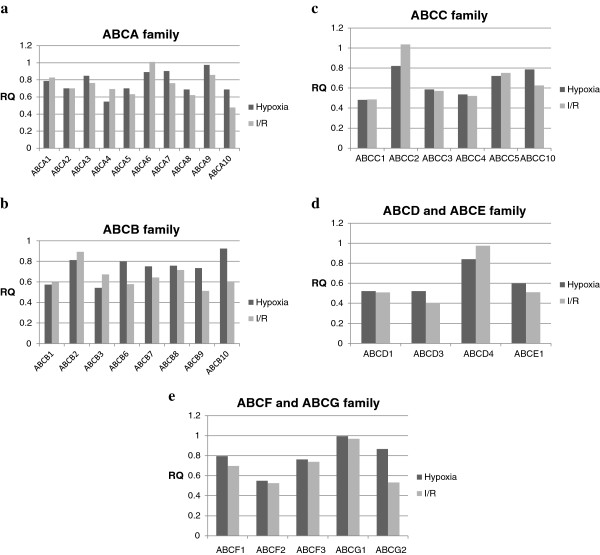
(**a-e**) **Expression and regulation of ABC transporters in the immortalised human brain microvascular endothelial cell line hCMEC/D3 after hypoxia and after ischemia/reperfusion compared to normoxic samples.**

Also displayed in Table 1 are RQ values of hCMEC/D3 gene expression compared to the human universal reference total RNA. Values are the mean (range). A high basal expression level, which was defined as a two-fold higher expression than in the pooled tissue RNA, is depicted in red. Low basal expression level, defined as two-fold lower expression than in the pooled tissue RNA, is depicted in blue. Most genes had high basal expression levels – some as high as up to almost 30-fold in the case of ABCB1 – and ABCA2, ABCA8 and ABCC2 revealed decreased basal expression levels. The expression levels of ABCA1, ABCA9, ABCA10, ABCB9, ABCG1, and ABCG2 were not changed. We could not determine a fold change value for ABCA7, because it could not be detected in the pooled tissue RNA. For an overview of RQ values, see also Figure 2.

### *Expression analysis of ABC-transporters in hCMEC/D3 cell line during hypoxia and hypoxia plus reoxygenation*

To compare the effects of ischemia or I/R on mRNA expression of human ABC transporters in the hCMEC/D3 cell line, we determined expression levels for samples which had undergone 24 hours of hypoxia and those who had undergone 24 hours of hypoxia and then 60 minutes of reoxygenation (I/R) to normoxic samples. For this analysis, we compared the fold change (RQ) in ABC transporter expression of hypoxic and I/R samples to normoxic samples (Table 2). Values are the mean (range). Genes that are regulated more than 2-fold (≥2.0 or ≤0.5) are considered as significantly regulated. Induced genes are depicted in red, down-regulated genes are depicted in blue.

**Table 2:** Expression and regulation of ABC transporters in the immortalised human brain microvascular endothelial cell line hCMEC/D3 after hypoxia and after ischemia/reperfusion

**Gene**	**RQ of hypoxic compared to normoxic samples (Range)**		**RQ of I/R compared to normoxic samples (Range)**	
ABCA1	0.79	(0.45-1.38)	0.83	(0.54-1.28)
ABCA2	0.70	(0.42-1.16)	0.70	(0.54-0.91)
ABCA3	0.85	(0.57-1.25)	0.76	(0.59-0.98)
ABCA4	0.55	(0.32-0.94)	0.69	(0.36-1.34)
ABCA5	0.70	(0.47-1.05)	0.63	(0.51-0.78)
ABCA6	0.89	(0.62-1.29)	1.01	(0.63-1.63)
ABCA7	0.90	(0.56-1.45)	0.76	(0.60-0.96)
ABCA8	0.69	(0.42-1.13)	0.62	(0.42-0.92)
ABCA9	0.97	(0.43-2.23)	0.86	(0.52-1.41)
ABCA10	0.69	(0.43-1.11)	0.48	(0.33-0.69)
ABCB1	0.57	(0.39-0.85)	0.60	(0.47-0.75)
ABCB2	0.81	(0.55-1.20)	0.89	(0.70-1.15)
ABCB3	0.54	(0.35-0.85)	0.67	(0.47-0.95)
ABCB6	0.80	(0.50-1.28)	0.58	(0.47-0.71)
ABCB7	0.75	(0.51-1.11)	0.64	(0.51-0.81)
ABCB8	0.76	(0.47-1.22)	0.71	(0.50-1.02)
ABCB9	0.74	(0.48-1.13)	0.51	(0.35-0.75)
ABCB10	0.92	(0.60-1.42)	0.60	(0.46-0.77)
ABCC1	0.48	(0.33-0.71)	0.49	(0.39-0.61)
ABCC2	0.82	(0.48-1.39)	1.03	(0.71-1.51)
ABCC3	0.59	(0.40-0.85)	0.57	(0.44-0.74)
ABCC4	0.54	(0.37-0.79)	0.52	(0.40-0.68)
ABCC5	0.72	(0.45-1.15)	0.75	(0.40-1.42)
ABCC10	0.79	(0.48-1.29)	0.63	(0.48-0.82)
ABCD1	0.52	(0.30-0.90)	0.51	(0.25-1.02)
ABCD3	0.52	(0.34-0.79)	0.40	(0.27-0.61)
ABCD4	0.84	(0.55-1.28)	0.98	(0.63-1.51)
ABCE1	0.60	(0.41-0.87)	0.51	(0.38-0.69)
ABCF1	0.80	(0.53-1.18)	0.70	(0.54-0.91)
ABCF2	0.55	(0.34-0.88)	0.53	(0.43-0.64)
ABCF3	0.76	(0.49-1.20)	0.74	(0.46-1.17)
ABCG1	0.99	(0.68-1.44)	0.97	(0.76-1.23)
ABCG2	0.87	(0.59-1.27)	0.53	(0.36-0.78)

No genes were induced under hypoxia or I/R. We observed a significant down-regulation for ABCA10 in I/R samples, but no further changes for mRNA in the ABCA family. The ABCA family is mainly involved in lipid transports. We did not discover any significant changes in the ABCB family, whose transporters functions in multi-drug resistance (ABCB1), mitochondrial activity (ABCB6, ABCB7, ABCB8, ABCB10) or lysosomal activity (ABCB9). Of the ABCC family, only mRNA levels of ABCC1, a multi-drug resistance protein, were decreased both in hypoxic and I/R samples. No significant changes were found for the other members of the family, which is predominantly involved in multi-drug resistance. We noted a down-regulation of ABCD3 in I/R samples, but no other expression alterations were significant in the ABCD, ABCE, ABCF and ABCG family. Members of the ABCD family are only found in peroxysomes and are involved in very long chain fatty acid oxidation. ABCE1, the only member of the ABCE family, inhibits the RNAseL protein and is essential for the assembly of immature human immunodeficiency virus capsids. ABCF transporters, like ABCF1, have no transmembrane domains and may play a role in enhancement of protein synthesis and the inflammation process. ABCG1 is involved in cholesterol efflux, ABCG2 is a drug-resistance gene. The other members of the ABCG family are mainly involved in cholesterol and sterol transport in various organs, but were not detected in hCMEC/D3 cell line. Overall, we noticed a general tendency towards down-regulation for all genes in hypoxic and I/R samples except for ABCA6 and ABCC2 of I/R samples (Table 2).

## Discussion

In this study, we have employed a TaqMan-based low-density array for mapping mRNA expression profiles in hCMEC/D3 cell line for 47 of the known 48 ABC transporters. We compared expression levels of normoxic hCMEC/D3 cells to hCMEC/D3 samples which had undergone 24 hours of hypoxia and to hCMEC/D3 samples which were reoxygenated for 60 minutes after 24 hours of sustained hypoxia (a model for I/R). Our main aim was to investigate, whether the developed hCMEC/D3 cell line [23] might be a reliable and accessible in vitro model to study the effects of ischemia upon ABC transporters. Surprisingly, we did not detect a significant up-regulation of any ABC transporter mRNA neither in hypoxic hCMEC/D3 nor in cells subjected to I/R.

Most of our results regarding overall expression of ABC transporter mRNA in normoxic samples are in concordance with previously published data for hCMEC/D3 cell line [25,36], for example the rather high expression of ABCB1, ABCB3 and ABCC1. Comparison is difficult though due to different parameters, housekeeping genes or calibrators [25,36]. Notable are the differences of expression in hCMEC/D3 cell line compared to expression levels in the adult human BBB: Dauchy et al. [37] showed no detection for ABCC2 and ABCC3 mRNA neither in cortex samples nor in the corresponding isolated microvessel fraction, both of which could be detected in our study in hCMEC/D3 cell line (Table 1). However, for ABCC2 the discrepancy may be attributed to the fact, that the hCMEC/D3 cell line is based on samples taken from epileptic regions [23] and ABCC2 expression is known to be found in brain samples from epileptic regions [38]. The known upregulation of several ABC transporters in samples of epileptic regions [39] might also be the cause for further controversies such as the rather high ABCC1 mRNA levels or the absence of genes in our study, for example ABCC6 and ABCC11 (Table 1), which were both detected in other studies [37,40]. Expression levels differed all the same: Dauchy et al. [37] found ABCG2 mRNA levels to be approximately sevenfold higher than ABCB1 levels, whereas in our study and in another study from Dauchy et al. [25] ABCG2 mRNA levels were approximately sevenfold lower than ABCB1 mRNA levels. These findings strongly suggest a significant contrast in expression patterns of ABC genes in hCMEC/D3 cell line to human BBB samples and underline findings of other studies, in which ABCB1, ABCC1 and ABCG2 mRNA expression differed greatly from expression levels in human brain microvessels [25].

Regarding the effects of hypoxia on the expression of ABC transporters, our findings indicate a tendency towards down-regulation for most ABC transporter genes in hypoxia, especially taking into consideration the RQ ranges (Table 2), but only ABCC1 was significantly down-regulated after 24 hours of hypoxia. There is very few data on the effects of hypoxia on ABC gene expression in brain endothelial cells in vitro: Only one recently published study from Patak et al. [24] investigated the effects of hypoxia on ABCC1 and ABCB1 gene expression, but found no change in hCMEC/D3 cell line after 4 hours of hypoxia (nor in protein levels after 4 to 48 hours of hypoxia for that matter). The difference in ABCC1 expression as well as the lower RQ of ABCB1 in our study (0.57 versus almost 1 in the study from Patak et al.) may be mainly attributed to the increased duration of hypoxia in our study and not to variance, since even RQ_max_ of ABCB1 did not reach 1 in our study. Three more studies focused on ABCB1 mRNA expression in rat brain endothelial cells: Xiao-Dong et al. [41] showed that repetitive/temporal hypoxia of 15 minutes once a day (by covering the cells in paraffin oil) over a span of 8 days induced increase of ABCB1 levels, Felix and Barrand [42] found increased levels of ABCB1 following 6 hours of hypoxia, Robertson et al. [43] found no changes after 6 hours of hypoxia alone. These results in rat brain endothelial cells are largely in contrast to our findings, where no change of ABCB1 levels could be detected. This may be due to the longer hypoxic duration in our study, leading to more cell death and general down-regulation of genes, but in synopsis with the aforementioned results from Patak et al. the different results are probably related to interspecies differences. Thus, comparison between results in rat brain endothelial cell lines and hCEMC/D3 cell line should be viewed critically.

In our samples that were reoxygenated for 60 minutes after 24 hours of hypoxia, we found significantly decreased expression levels for ABCA10, ABCC1 and ABCD3, but no significant changes for other genes. The mRNA levels of hypoxic cells after reoxygenation tended to decrease slightly more as compared to hypoxic cells without reoxygenation. These findings suggest that reoxygenation results in additional cell stress.

Compared to the results of in vivo studies, which investigated the expression of ABC transporters after ischemia, our results were controversial: We did not find any elevated expression of mRNA levels neither after 24 hours of hypoxia nor after 24 hours of hypoxia followed by 60 minutes of reoxygenation. However, ABCB1 proved to be upregulated on focal cerebral ischemia in mice [39], but not in rats [44], whereas ABCC8 showed *de novo* expression on ischemia in rats [39]. ABCC5 and ABCG2 showed elevated expression after ischemia in a rat model for stroke [44].

Differences in cerebral endothelial cell gene expression from in vivo models and non-immortalised rat and mouse brain endothelial cell cultures to our results can be seen as an interspecies difference, but may also be discussed from the perspective of immortalisation: hCMEC/D3 cell line is immortalised by use of hTERT and SV 40 large T antigen. hTERT adds a functional telomerase to hCMEC/D3 cells, SV 40 large T antigen deactivates p53 and pRb, thus inhibiting apoptosis and facilitating cell proliferation. It seems only plausible, that a cell line altered in such a way is more resilient when it comes to oxygen deprivation and/or oxidative stress, forcing longer durations of hypoxia in order to observe significant changes than one would need in primary cells. Changes of mRNA expression after hypoxia observed in vivo could also be attributed to other cells than endothelial cells, for example glial cells or neurons, and discussed in the interaction within the neurovascular unit [5,45]. Interestingly, Dazert et al. [44] only found significant mRNA upregulation from days 3–14, but not earlier, suggesting that upregulation of ABCG2 and ABCC5 is rather linked to behavioural recuperation than an imminent effect of hypoxia. Further investigation differentiating between short-term and mid-term effects of hypoxia and the effect of neuro-endocrine crosstalk i.e. with ischemic astrocytes within the concept of the neurovascular unit upon the mRNA expression levels of cerebral endothelial ABC transporters may help to better characterise ischemia and I/R at the BBB [46].

The study described here, however, has some limitations, and duration of hypoxia in our hypoxia set up in relation to HIF-1α expression and therefore changes in ABC transporter expression has not been performed to date. Expression of HIF isoforms may differ, and Patak et al. showed that in hCMEC/D3 during hypoxia (at 0 and 1 % oxygen) HIF-1α and HIF-2α abundance increased within 4 h. HIF-1α levels then decreased to below detection levels within 16 h of hypoxia and HIF-2α remained elevated even after 48 h. The complex relationships of HIF expression and changes in ABC transporter expression may be investigated further, but correlation to the situation in ischemic stroke may remain critical.

Our findings prove significant for clinical aspects of ABC transporters: Increased mRNA levels of ABCC8 for example play an important role in genesis of cerebral edema after ischemic stroke [47]. Early administration of glibenclamide appears to be a potent treatment to reduce vasogenic brain edema [48]. ABCG2 among other ABC transporters proved to be upregulated after an ischemic insult in order to prevent the crossing of toxic compounds over the BBB and might positively affect neurogenesis [44]. ABCB1, which limits the access of unwanted substrates to the brain, shows increased expression under oxidative stress, as it occurs during I/R [42] and nitric oxide might contribute to this up-regulation [49]. The hCMEC/D3 cell line however did not show elevated expressions for either ABCB1 or ABCG2 and no expression at all for ABCC8, depriving us of the opportunity to study the clinical importance of ABC transporters under hypoxia or after I/R in an easily accessible model.

## Conclusion

We report that the regulation of ABC transporters in case of ischemic stroke could not be reproduced in vitro using the hCMEC/D3 cell line as a stroke model. Our findings on ABC-transporter expression at cerebral endothelial cells using the hCMEC/D3 cell line differ largely from previously published in-vivo and in-vitro studies. Although the hCMEC/D3 cell line recapitulates most of the unique properties of the blood–brain barrier and is an excellent tool to study transendothelial transport [23] it may not be suitable for studying endothelial cell response to ischemia. This is in concordance with the findings of Balser from our group (data unpublished), who investigated the effects of hypoxia and repetitive exposition of isoflurane and sevoflurane on the expression of eNOS and iNOS and on the output of NO in hCMEC/D3 cell line and found no significant changes as opposed to studies on other (cerebral) endothelial cells. Furthermore, as Patak et al. noted, the regulation of ABC transporters seen in cerebral ischemia could depend on other factors than hypoxia alone such as glucose depletion or reactive oxygen species that are generated by reoxygenation [24]. At the moment, in vitro models in general form a poor basis for stroke research but may be enhanced by adding more cells of the neurovascular unit [46].

## Competing interests

The authors declare that they have no competing interests.

## Authors’ contributions

CL carried out the cell culture experiments, RNA isolation and drafted the manuscript. FW assisted with the cell culture and RNA isolation. AS performed the rt-PCR experiments and helped to analyse the data. FS designed the study, evaluated the data and corrected the draft. GS, UB and PC made valuable revisions of the manuscript. All authors read and approved the manuscript.
